# ***γ***-Secretase Inhibitor Alleviates Acute Airway Inflammation of Allergic Asthma in Mice by Downregulating Th17 Cell Differentiation

**DOI:** 10.1155/2015/258168

**Published:** 2015-08-03

**Authors:** Weixi Zhang, Xueya Zhang, Anqun Sheng, Cuiye Weng, Tingting Zhu, Wei Zhao, Changchong Li

**Affiliations:** ^1^Department of Pediatric Pulmonology, The Second Affiliated Hospital & Yuying Children's Hospital of Wenzhou Medical University, 109 Xueyuan Road, Wenzhou, Zhejiang 325027, China; ^2^Division of Allergy and Immunology, Department of Pediatrics, Virginia Commonwealth University, P.O. Box 980225, Richmond, VA 23298, USA

## Abstract

T helper 17 (Th17) cells play an important role in the pathogenesis of allergic asthma. Th17 cell differentiation requires Notch signaling. *γ*-Secretase inhibitor (GSI) blocks Notch signaling; thus, it may be considered as a potential treatment for allergic asthma. The aim of this study was to evaluate the effect of GSI on Th17 cell differentiation in a mouse model of allergic asthma. OVA was used to induce mouse asthma model in the presence and absence of GSI. GSI ameliorated the development of OVA-induced asthma, including suppressing airway inflammation responses and reducing the severity of clinical signs. GSI also significantly suppressed Th17-cell responses in spleen and reduced IL-17 levels in serum. These findings suggest that GSI directly regulates Th17 responses through a Notch signaling-dependent pathway in mouse model of allergic asthma, supporting the notion that GSI is a potential therapeutic agent for the treatment of allergic asthma.

## 1. Introduction

Asthma is an allergic disease characterized by airway inflammation, mucin hypersecretion, and airway hyperresponsiveness (AHR) [1]. Infiltration of CD4^+^ T cells, eosinophils, mast cells, and B cells and their interaction with airway resident cells contribute to airway inflammation [2]. IL-17-producing (Th17) CD4^+^ Th cells are important players in asthma pathogenesis [3, 4]. Numerous studies have shown an increase of Th17 cells in inflammatory airway [5, 6]. Therefore, suppressing Th17 response may be a novel therapeutic strategy for treating asthma. Notch signaling pathway is evolutionarily conserved. In mammalian, there are four Notch receptors (Notch1, Notch2, Notch3, and Notch4) and five Notch ligands (Jagged1, Jagged2, Delta-like ligand (Dll)1, Dll3, and Dll4) [7]. Notch plays a crucial role in a broad spectrum of cellular activities such as proliferation, differentiation, and regulation of cell function [8]. Notch signaling is initiated when Notch receptors are engaged with a Notch ligand. A series of enzymatic reactions lead to the cleavage of the Notch receptor intracellular domain (NICD) which is translocated to the nucleus, where it binds with CSL/RBP-Jk to recruit Mastermind-like 1 protein. The newly formed complex then initiates the transcription of downstream genes.

Previously, we showed that Notch signal pathway regulates the proliferation and differentiation of CD4^+^ T lymphocytes in a mouse model of asthma, indicating that Notch may be a potential target for treating asthma [9]. Others have reported that pharmacologic inhibitor of Notch signaling can reduce allergic pulmonary inflammation by modulating Th1 and Th2 responses [10, 11]. However, the exact protective mechanism of Notch signaling in asthma remains unknown. The present study aims to test whether GSI has therapeutic effects on the development of asthma through regulating Th17 mediated immune response.

## 2. Materials and Methods

**Figure 1:**
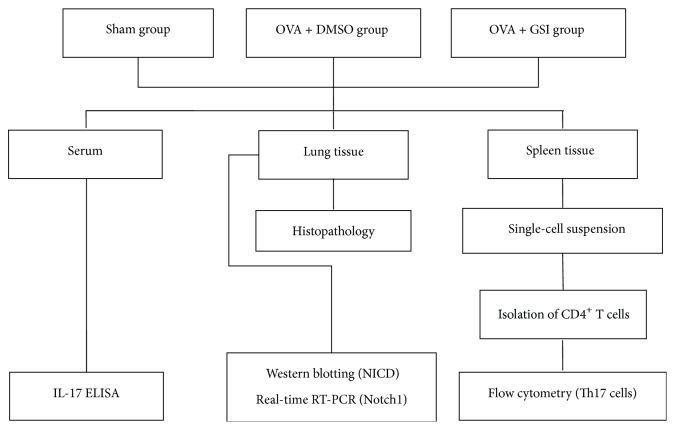
Flowchart of experimental design.

Experimental design is outlined in Figure 1.

### 2.1. Animal Model of Asthma

Male BALB/C mice, 4 to 6 weeks old, weighing 20–22 g, were purchased from Shanghai Laboratory Animal Center (Shanghai, China) and bred in pathogen-free environment in the animal center of Wenzhou Medical University. Animal experimental protocol was approved by Institutional Animal Care and Use Committee (IACUC) of Wenzhou Medical University. OVA induced asthma was established as described previously [9]. Experimental animals were divided into three groups: sham group, OVA + DMSO (vehicle) group, and OVA + GSI (0.3 mg/kg) group. Mice were sensitized by i.p. injection of 10 *μ*g ovalbumin (OVA) (Sigma, USA) emulsified in 20 mg Al (OH)_3_ gel in 0.1 mL normal saline (NS) on days 1 and 13. They were then challenged with OVA (1 mg/mL) aerosol for 30 min daily for eight consecutive days from day 25 by Jet nebulizer (Pari IS-2 Jet nebulizer; PARI Respiratory Equipment). GSI L685,458 (Calbiochem, CA) was administered intranasally 30 minutes before each OVA challenge at 0.3 mg/kg as previously described [11]. The sham mice were sensitized and challenged with normal saline (NS) and treatment with dimethylsulfoxide (DMSO) as a control for GSI. Mice were sacrificed within 24 h after the last allergen challenge.

### 2.2. Histopathological Examination

At the time of sacrifice, the left lung tissue was first fixed with 4% paraformaldehyde for 4 h. It was then dehydrated in ethylic alcohol, embedded with paraffin, sectioned in 4 *μ*m, and stained with haematoxylin and eosin (HE). Tissue slices were evaluated through light microscope (Nikon) by trained technician in a blind fashion.

The degree of allergic airway inflammation was scored according to the following histologic grading system (scored 0–4): absence of peribronchial inflammatory cells; a few scattered peribronchial inflammatory cells involving less than 25% of the circumference of the bronchus; focal peribronchial inflammatory cells infiltration not completely surrounding a bronchus (i.e., involving approximately 25%–75% of the circumference of the bronchus); one definite layer of peribronchial inflammatory cells completely surrounding a bronchus; 2 or more layers of peribronchial inflammatory cells completely surrounding a bronchus. In each lung section the mean peribronchial inflammatory score was determined by the sum of scores of all individual bronchioles in the section divided by the number of bronchioles [12].

### 2.3. Preparation of Splenic Single-Cell Suspension

The spleen tissue was fragmented into small pieces that were then pressed against nylon mesh with a plunger of a disposable syringe. Erythrocytes were lysed by red blood cell lysis buffer. Cells were washed in PBS.

### 2.4. Isolation of CD4^+^ T Cells

CD4^+^ T cells from splenic single-cell suspension were isolated by magnetic cell sorting by positive selection method using mouse CD4^+^ T cell isolation kit (MACS, Miltenyi Biotec, Germany) according to the manufacturers' instruction. The purity of the cells was 92.04 ± 5.18%, as confirmed by flow cytometry analysis.

### 2.5. Flow Cytometry Analysis

FITC-labeled anti-mouse CD4 and PE-labeled anti-mouse IL-17A were used to detect Th17 cells. Matching IgG was used as isotype control. All antibodies were purchased from BD Bioscience, USA.

For Th17 cell analysis, CD4^+^ T cells (1 × 10^6^/mL) from the spleen tissue were stimulated for 4.5 hr with phorbol myristate acetate (PMA) at 100 ng/mL and Ionomycin at 1 *μ*g/mL in the presence of 1.6 *μ*g/mL Monensin (all from Beyotime, China). Cells were collected, washed, and surface-stained with FITC-labeled anti-CD4 antibody at 4°C for 20 min in the dark and resuspended in Fix/Perm solution according to the manufacturer's instruction (Invitrogen, USA). They were then stained intracellularly with PE-labeled anti-IL-17 antibody. After washing, cells were resuspended in fixation solution and subjected to FACScalibur flow cytometer (BD FACSCanto II, USA) analysis. Background fluorescence was assessed by the corresponding isotype control antibodies. Data were analyzed with WinMDI software.

### 2.6. Western Blotting

Lung tissue was fragmented and lysed in RIPA buffer with protease inhibitor mixture (Beyotime, China). A total protein of 20 *μ*g was loaded into each well of a SDS-PAGE gel for separation by electrophoresis and then transferred onto nitrocellulose membrane. The resulting blots were blocked for 1 h with TBS Tween 20 containing 5% powder skim milk and then probed overnight at 4°C with anti-NICD (Abcam, UK). Blots were then washed three times and probed for 1 h with anti-rabbit HRP-conjugated antibody. *β*-actin mouse mAb (Beyotime, China) was used as the loading control. Immunostained proteins were detected by ECL.

### 2.7. Quantitative Real-Time RT-PCR Analysis

RNA was extracted from lung tissue using Trizol (Invitrogen, USA) according to the manufacturer's instruction. cDNA was synthesized by reverse transcription with oligo (dT) from total RNA. The quantitative real-time RT-PCR was performed using an ABI Step One Plus System (Applied Biosystems, USA) with QuantiFast SYBR Green PCR Kit (QIAGEN, Germany). GAPDH was used as internal control. Primers used were as follows: GAPDH, 5′-TGGCCTTCCGTGTTCCTAC-3′ (forward) and 5′-GAGTTGCTGTTGAAGTCGCA-3′ (reverse); Notch1, 5′-TGCCACAATGAGATCGGCTC-3′ (forward) and 5′-GAGTTGCTGTTGAAGTCGCA-3′ (reverse). Delta-Delta Ct method was used to express the fold induction of target mRNA after GAPDH normalization.

### 2.8. ELISA

The concentration of cytokines IL-17 in serum was assessed by standardized sandwich ELISA according to the manufacturer's protocol. The IL-17 kit was purchased from eBioscience, San Diego, USA.

### 2.9. Statistical Analysis

All data were expressed as Mean ± SEM. Differences between groups were analyzed for statistical significance by one-way analysis of variance using SPSS 13.0 software (SPSS Inc., Chicago, USA). Differences with a *P* value < 0.05 were considered statistically significant.

## 3. Results

### 3.1. GSI Ameliorates the Severity of Inflammation in OVA-Induced Asthma

**Figure 2:**
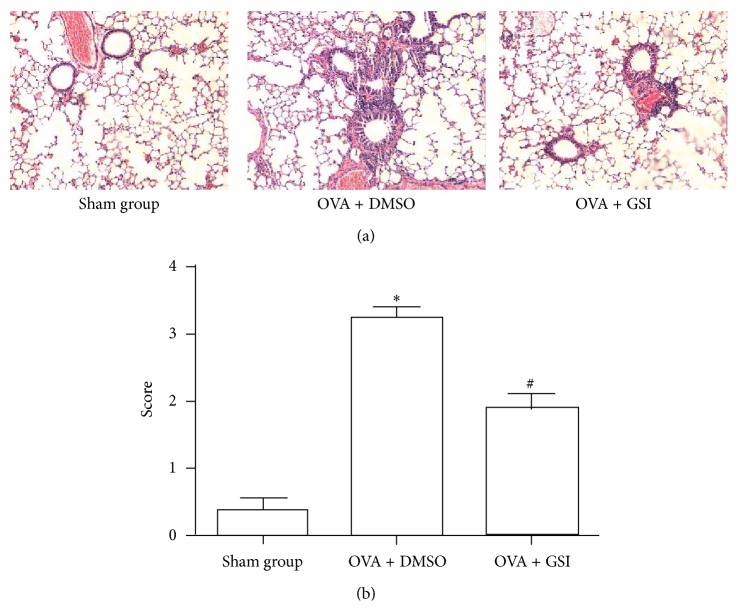
*γ*-Secretase inhibitor (GSI) reduced OVA-induced airway inflammation. (a) BALB/C mice were sensitized i.p. with OVA and challenged with OVA in the presence and absence of GSI. Mice were sacrificed within 24 hr after last challenge. Lung tissues were stained with haematoxylin and eosin and subjected to light microscope (×200) examination. (b) Semiquantitative pathology scores among sham, OVA, and OVA + GSI groups. Data expressed as Mean ± SEM. *N* = 8 mice per group. ^*∗*^ *P* < 0.01 compared with the sham group; ^#^ *P* < 0.01 compared with the vehicle group.

To explore the effect of GSI on OVA-induced asthma, BABL/C mice were sensitized and challenged with OVA (or NS for sham mice). Those mice received GSI, a highly selective inhibitor of *γ*-secretase or vehicle (DMSO), during the challenge phase. Mice were sacrificed within 24 h after the last allergen challenge and lung tissue was fixed, embedded, and sectioned for HE staining. The degree of airway inflammation of HE-stained lung tissue was scored as described in Materials and Methods. As shown in Figure 2(a), OVA + DMSO group demonstrated significant infiltration of eosinophils and lymphocytes with marked thickening of airway wall and epithelial goblet cell metaplasia, as compared with the sham group. GSI treatment reduced such inflammation and airway wall thickening (Figure 2(b)). OVA-challenged mice showed an inflammation score of 3.25 ± 0.46 as compared to sham control (0.38 ± 0.52). GSI treated group showed a score of 1.88 ± 0.64 that is significantly lower than OVA-DMSO group (Figure 2(b), *P* < 0.01).

### 3.2. GSI Affects the Expression of Notch Signaling Component

**Figure 3:**
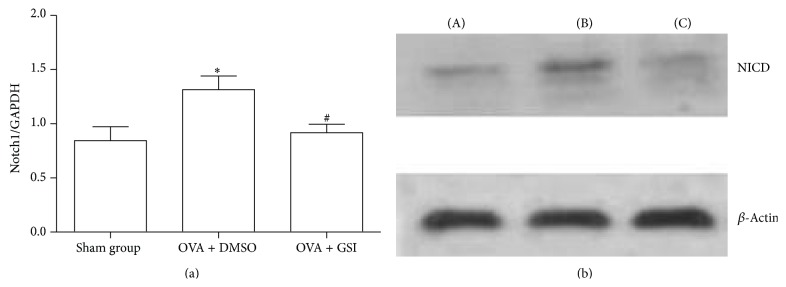
GSI decreased Notch1 and NICD. BALB/C mice were sensitized i.p. with OVA and challenged with OVA in the presence and absence of GSI. (a) The expression of Notch1 mRNA was evaluated by quantitative real-time RT-PCR. GAPDH was used as internal control. (b) Protein levels of NICD were examined by Western blotting. *β*-actin was used as a loading control. (A) Sham group; (B) OVA + DMSO; (C) OVA + GSI. Data expressed as Mean ± SEM. *N* = 8 mice per group. ^*∗*^ *P* < 0.01 compared with the sham group. ^#^ *P* < 0.01 compared with the vehicle group.

To investigate the blockage effects of GSI on Notch signaling, mRNA expression of Notch1, a receptor of Notch signaling, was examined. As shown in Figure 3(a), OVA-challenged mice revealed enhanced Notch1 mRNA expression, as compared with the sham group (1.31 ± 0.13 versus 0.84 ± 0.13, *P* < 0.01). On the other hand, GSI treatment led to the reduction of Notch1 mRNA expression (0.92 ± 0.088  *P* < 0.01 comparing to OVA group). Consistent with this observation, OVA-challenged mice revealed increased NICD generation as compared to sham group (0.18 ± 0.02 versus 0.09 ± 0.01, *P* < 0.01). GSI treatment decreased NICD generation (0.06 ± 0.03) comparing to OVA group (Figure 3(b), *P* < 0.01). Results presented here suggest that GSI can effectively block Notch signaling.

### 3.3. GSI Decreases the Frequency of Th17 Cells in the Spleen of OVA-Induced Asthma Mice

**Figure 4:**
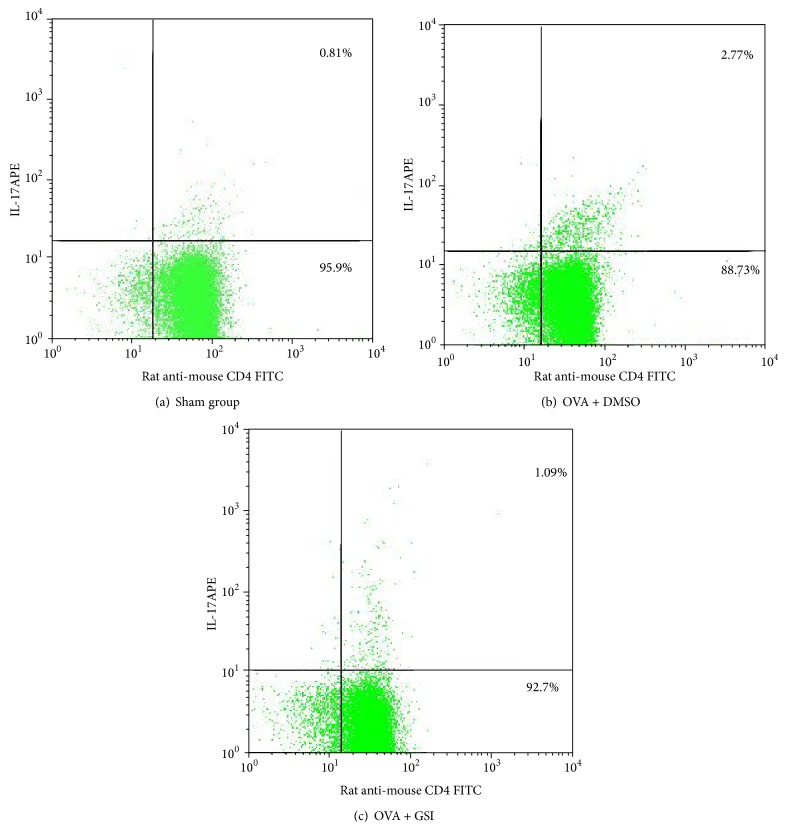
GSI administration resulted in reduced Th17 cell expansion. BALB/C mice were sensitized i.p. with OVA and challenged with OVA in the presence and absence of GSI. Splenic CD4^+^ T cells were isolated by magnetic cell sorting. Th17 cells were examined by IL-17A staining and data were analyzed by flow cytometry. Dot plots show as percent of cells positive for CD4 and IL-17A staining. Graphs representative of one of eight experiments.

To evaluate the effect of GSI treatment on Th17 cell expansion, splenic CD4^+^ T cells were isolated by magnetic cell sorting. Th17 cells were identified by IL-17A staining. Sham group expressed a baseline Th17 cell frequency of 0.30 ± 0.16% of total splenic CD4^+^ T cells. OVA-induced asthma mice revealed a significant increase of Th17 cells (2.43 ± 0.69%, *P* < 0.01, comparing to sham group). GSI treatment reduced Th17 cell frequency to 1.26 ± 0.85% which is statistically significant from OVA group (*P* < 0.05, Figure 4). This finding indicates that GSI reduces the development of Th17 cells.

### 3.4. GSI Treatment Reduces the Production of IL-17 of Asthma Mice

**Figure 5:**
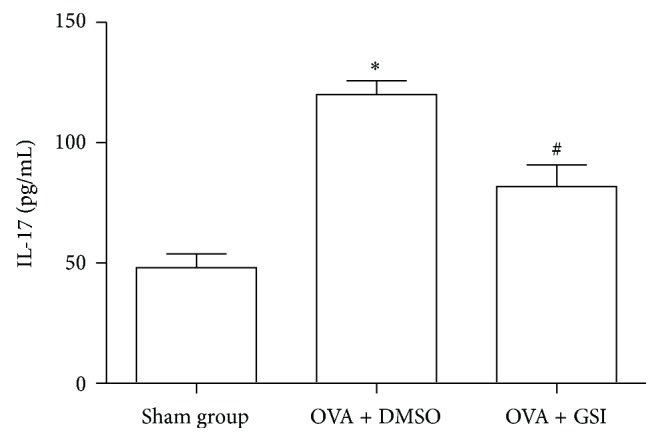
GSI administration reduced production of IL-17. Serum IL-17 levels were measured from sham, OVA, and OVA plus GSI groups using standardized sandwich ELISA. Data expressed here are Mean ± SEM. *N* = 8. ^*∗*^ *P* < 0.01 compared with the sham group. ^#^ *P* < 0.01 compared with the vehicle group.

Th17 cells are the main source of IL-17. To further examine the function of such Th17 cells, serum levels of IL-17 were measured from OVA-induced asthma mice. As illustrated in Figure 5, sham group expressed a baseline level of IL-17 in serum at 48.07 ± 5.73 pg/mL. The IL-17 level was significantly elevated in OVA-induced asthma mice (120.09 ± 5.73 pg/mL, *P* < 0.01). GSI administration during challenge phase significantly reduced the IL-17 level to 81.82 ± 8.95 pg/mL, *P* < 0.01. These findings confirm the possibility that GSI downregulates IL-17 expression.

## 4. Discussion

The Notch signaling pathway is involved in many aspects of organ formation and cell function [7]. Dysregulation of Notch signaling may induce human disorders such as asthma. The effect of Notch signaling inhibition on the development of asthma has been addressed in several recent studies. Jin and colleagues reported that inhibition of Notch signal pathway by GSI alleviated the airway inflammation in OVA-induced asthma model and it was through the regulation of Th1 and Th2 responses [11]. Knockdown of the Notch l gene by small interfering RNA led to overproduction of IL-4 and IFN-*γ*, which played an important role in the pathogenesis of asthma [13]. However, the exact underlying mechanism is yet to be fully elucidated.

In the present study, we used GSI to block Notch signaling in a mouse model of asthma and demonstrated that in vivo administration of GSI effectively attenuated eosinophilic and lymphocyte infiltration in the airways and decreased goblet cell metaplasia. Furthermore, Notch inhibition by GSI reduced the frequency of Th17 cells in spleen and the serum levels of IL-17. Taken together, these findings demonstrate the effectiveness of GSI in animal models and strongly suggest that inhibition of Notch signaling could be an effective strategy for the treatment of asthma.

T helper 17 (Th17) cells play a critical role in adaptive immune responses through the production of cytokines, namely, IL-17A, IL-17F, and IL-22 [14]. Recent studies indicate that Th17 cells are active players in acute airway inflammation of allergic asthma. Marked elevation of IL-17A was detected in the sputum of severe asthma patients [15]. Excess IL-17-secreting cells were observed in the lung tissue of such patients as well [16]. Li et al. [17] suggested that the ratio of Th17 cells/CD3^+^ T cells in peripheral blood was significantly increased in asthma patients compared with nonasthma individuals. Similar change was found in the present study; that is, the proportion of Th17 cells in isolated spleen CD4^+^ T cells was significantly increased in OVA-induced asthma mice compared to the sham group. McKinley et al. reported that adaptive transferring of Th17 cells to an asthma mouse led to neutrophils infiltration and airway hyperresponsiveness, which is resistant to corticosteroid therapy [18]. IL-17 mediated airway inflammation may result in severe airway obstruction. The expression of IL-17A was also noticed in ozone exacerbated asthma [19]. Consistent with these findings, our investigation revealed that treatment with GSI markedly reduced serum IL-17 levels of asthma mice. Of note, Doe et al. [20] found that IL-17A was elevated in mild to moderate human asthma compared with healthy control, while it was not increased in severe asthma. Therefore, further research is needed to characterize the exact relationship between IL-17 and the severity of asthma.

Notch can directly regulate retinoic acid-related orphan receptor *γt*, an important transcription factor for Th17 differentiation [21]. The effect of GSI on autoimmune inflammatory disorders has been addressed in several studies. *γ*-Secretase inhibitor treatment can downregulate Th17 response and inhibit vascular inflammation [22]. Inhibition of Notch signaling by Notch3 antibody attenuates Th17-type responses, while treatment with Notch ligand Delta-like 1 promotes Th17 response [23]. In vitro knockdown of Notch and in vivo administration of GSIs result in reduced IL-17 production and substantially impede Th17-mediated disease progression in mouse model of multiple sclerosis [24]. GSIs have been actively tested in clinical trials for Alzheimer disease for their potential in blocking the generation of A peptide [25]. MRK003, a *γ*-secretase inhibitor, exhibits promising in vitro preclinical activity in multiple myeloma and non-Hodgkin's lymphoma [26]. After blocking Notch signaling in our mouse asthma model, we noticed decreased level of NICD, ameliorated airway inflammation, reduced serum IL-17 level, and improved clinical signs. Our data strongly suggest that inhibition of Notch signaling could be considered as an effective therapy for asthma. Of course, further preclinical and clinical research is needed to address such potential.

In conclusion, the current study proves that GSI administration inhibits Th17 differentiation, decreases IL-17 production, and alleviates airway inflammation in OVA-sensitized and OVA-challenged BALB/C mice. These results support the idea of considering GSI as a novel, effective antiasthma agent.

## Abbreviations

Th: T helper
GSI: *γ*-Secretase inhibitor
AHR: Airway hyperresponsiveness
Dll: Delta-like ligand
NICD: Notch receptor intracellular domain
IACUC: Institutional Animal Care and Use Committee
NS: Normal saline
DMSO: Dimethylsulfoxide
HE: Haematoxylin and eosin
PMA: Phorbol myristate acetate
OVA: Ovalbumin
Th17: IL-17-producing CD4^+^ Th cells.
